# Persisting neurobehavioral effects of developmental copper exposure in wildtype and metallothionein 1 and 2 knockout mice

**DOI:** 10.1186/s40360-016-0096-3

**Published:** 2016-11-02

**Authors:** Ann Petro, Hannah G. Sexton, Caroline Miranda, Anit Rastogi, Jonathan H. Freedman, Edward D. Levin

**Affiliations:** 1 Department of Psychiatry and Behavioral Sciences, Duke University Medical Center, Box #104790, Durham, NC 27710 USA; 2 Department of Neurology, Weill Cornell Medical College, Cornell University, New York, NY USA; 3 Department of Radiology, University of Florida College of Medicine, Gainesville, FL USA; 4 Department of Pharmacology and Toxicology, University of Louisville School of Medicine, Louisville, KY USA

**Keywords:** Copper, Metallothionein, Memory, Developmental, Radial-arm maze, Dopamine, Serotonin, Norepinephrine, Mouse

## Abstract

**Background:**

Metallothioneins (MT) are small proteins, which are crucial for the distribution of heavy and transition metals. Previously, we found in mice that knockout of MT 1 and 2 genes (MTKO) impaired spatial learning and potentiated the learning impairment caused by developmental mercury exposure. The current study examined the neurocognitive and neurochemical effects of MTKO with the developmental copper (Cu) supplementation.

**Methods:**

Wildtype (WT) and MTKO mice were given supplemental Cu (0, 10 or 50 mg/l) in their drinking water during gestation and until weaning. When the mice were young adults they were trained on the win-shift 8-arm radial maze test of spatial learning and memory. After cognitive testing, their brains were analyzed for norepinepherine, dopamine and serotonin levels.

**Results:**

In the spatial learning test, wildtype mice showed the normal sex difference with males performing more accurately than the females. This effect was eliminated by MTKO and restored by moderate Cu supplementation during development. In neurochemical studies, MTKO caused a significant overall increase in serotonin in all of the regions studied: the frontal cortex, posterior cortex, hippocampus, striatum, midbrain, and brainstem. MTKO also caused a significant increase in norepinepherine in the brainstem and hippocampus. In wildtype mice, Cu supplementation during development caused a significant decline in dopamine and norepinepherine in the midbrain and dopamine in the frontal cortex. These effects were blocked by MTKO.

**Conclusions:**

The normal sex difference in spatial working memory accuracy, which was eliminated by MTKO, was restored by moderate copper supplementation. MTKO increased serotonin across all brain areas studied and increased norepinepherine only in the hippocampus and brainstem. MTKO blocked copper-induced decreases in dopamine and norepinepherine in the midbrain and dopamine in the frontal cortex.

## Background

Metallothionein (MT) is a cysteine-rich, low molecular weight intracellular protein system whose function is to uptake, transport, and regulate transition metals, such as copper (Cu), throughout biological systems [1]. In doing so, MT maintains an optimal amount of essential metals and at the same time helping rid the body of non-essential potentially toxic metals. We have previously found in mice that knockout of MT 1 and 2 genes (MTKO) impaired spatial learning and potentiated the learning impairment caused by developmental mercury exposure [2, 3]. The influence of MT manipulations on reaction to alterations in essential metals such as Cu and neurocognitive function has not been well characterized.

Cu is essential metal for normal neuronal physiology. A dietary intake of 1–2 mg of Cu per day is considered essential for humans [4]. Potential sources of Cu exposure include drinking water, industry emissions, cooking utensils, fertilizers, bactericides, fungicides, algicides, and mineral rich foods such as vegetables, legumes, nuts, grains, fruits, and systems, and chocolate. Cu shows a typical developmental change with Cu concentration in mouse brain increasing by 200 % between 10 and 20 days after birth [5]. Changes in Cu levels have direct effects on monoaminergic transmitter systems. Dietary Cu deficiency in mice show a reduction in norepinepherine and dopamine concentrations [6].

Abnormal levels of Cu exposure, in the form of deficiency or excess, have been linked to neural dysfunctions. Cu crosses the placental barrier and when pregnant mothers are exposed to above normal Cu levels, the fetal development of the central nervous system is adversely affected [7]. Cu supplementation during infancy has been associated with an increased risk of Cu toxicity [8]. Importantly, biological reactions to Cu exposure vary person to person on a genetic basis. For example, some individuals are genetically predisposed to abnormal Cu metabolism and thus abnormal bodily Cu exposure. Menkes’ disease is an X-linked genetic disorder characterized by various mutational forms of the ATP7A gene and is associated with Cu accumulation in some tissues and simultaneous Cu deficiency in blood vessels and the brain [9, 10]. Neural degeneration and premature death are characteristic of this disorder. Wilson’s is an autosomal recessive genetic disorder consisting of mutations to the ATP7B gene, which leads to the release of free Cu into the bloodstream, causing toxic damage to several organs, including the brain [10]. The disease results in death if it goes untreated [1]. In addition, links have been made between Cu metabolism and several neurodegenerative disorders including Parkinson’s and Alzheimer’s disease [11].

Cu levels in the central nervous system are influenced by Cu intake during perinatal development. Cu deficiency during development can produce long-term functional neurobehavioral impairment [12]. Dietary Cu deficiency in mice during late gestation lowered norepinephrine concentrations in most brain regions. Dopamine in mice that had Cu deficiency during late gestation was elevated in cerebellum, medulla, hypothalamus and midbrain, but unchanged in cerebrum and striatum. Cu repletion normalized alterations in brain norepinephrine and dopamine concentrations. These data extend previous observations and suggest that persistent changes to brain may occur following perinatal Cu deficiency. These data also support the hypothesis that there is brain-regional specificity in response to Cu deficiency and repletion [13].

MT plays a significant role in the modification of Cu kinetics and toxicity, and is necessary for proper Cu metabolism. Cu is absorbed by intestinal cells and later by liver cells within which metallothioneins form non-toxic complexes with Cu, leading to Cu storage [10]. Cu that does not bind to MT is released into the blood or into the biliary canaliculi. MT’s significant role in Cu metabolism is also confirmed by its apparent role in genetic disorders of abnormal Cu metabolism. Menkes’ disease has been associated with disrupted metallothionein binding [9, 10]. The fact that Cu accumulation in some tissues in Menkes’ disease is found in the form of Cu-MT complex suggests that the disorder leads to or is associated with the dysregulation of MT gene expression [14]. It has also been demonstrated that MT is present in the placenta where it modulates metal transfer from mother to fetus [15, 16].

MT’s effects in relief of oxidative stress may be related to its role in protecting cells, tissues, and organisms from environmental chemical exposure [17]. This ability to alleviate oxidative effect may also serve to protect against neural damage due to toxic insult and aging. MT-1 and MT-2 protect the nervous system from physical injury and various compounds including interleukin 6, 6-aminonicotinamide, and kainic acid [18–20]. In addition, research has found that MT has a role in spatial learning and memory [3]. It has also been shown that MT is involved in neurobehavioral development and cognitive performance [21]. This study indicated that MT knockout mice were more susceptible to the neurobehavioral effects of heavy metal exposure in utero. In a previous study, we found that knockouts of MT 1 and 2 genes led to a developmental neurobehavioral expression of mercury metal toxicity [2].

In order to study the effects of Cu on cognitive-behavioral development and the possible modifying effects of MT in developmental neurotoxicity, two lines of mice were tested on radial-arm maze acquisition: wildtype and MT-1/MT-2 knockout mutants, both of which were exposed to varying levels of Cu in utero. The transgenic mice that had both MT1 and MT2 genes knocked out were produced following a past protocol using alternative heavy metals [22]. These mice do not demonstrate abnormal phenotypes except for lower levels of zinc in serum and liver [23, 24], learning impairment [3], and enhanced sensitivity to metal toxicity and environmental stress [25]. We hypothesized that the condition of metallothionein knock out mice exposed to Cu in utero would be characterized by enhanced cognitive impairment. By altering metallothionein genes, which are responsible for Cu metabolism, this study aims to demonstrate the resultant developmental neurobehavioral effects. Thus, the study provides an additional observation of genetically altered biological response to Cu exposure, as previously seen in Menke’s disease and Wilson’s disease.

The current project was conducted to determine the persisting neurobehavioral effects of MT 1 and 2 knockout and whether Cu supplementation during development would reverse the impairments in cognitive function as well as monoaminergic systems important for cognitive function.

## Methods

### Mice: genotype and Cu exposure

MT-1/MT-2-knockout mice and the control wild type mice were from the parental 129 strain provided by Jackson Labs. The knockout mice were homozygous for the Mt1tm1Bri Mt2tm1Bri mutation, having been produced by homozygous x homozygous matings. Both metallothionein genes had been knocked out during a single targeting event. The 129-derived AB-1 ES cell line was used and the knockout mice had been back-crossed to the 129 line for more than 22 generations.

Males and females, one pair per cage, at 8 weeks of age were mated. Females age 8 weeks or greater were assigned to 0, 10ug/L or 50ug/L Cu treatment. The Cu was supplied as Cu sulfate (Sigma, St. Louis, MO, USA) and the solutions were prepared as the base weight in acidified water (pH 4). The females were exposed to the Cu treatment for a minimum of 2 weeks prior to mating. Females in the 0 μg/L condition were given acidified water. For mating, the females were placed on water and a male was introduced and allowed to remain in the cage overnight. The following morning the male was removed and the female returned to the appropriate Cu treated water. Thus, the male was never exposed to Cu and the female was without Cu treatment for a period of approximately 16 h. The weight of the females was monitored during pregnancy. The pups remained with the mother until weaning at age 4 weeks. The pups were then given tap water and food ad lib. Litters with 3 or fewer animals were not used. The doses were chosen in light of effective dosages in the literature [7]. Thus, the study’s mice were exposed to various Cu water concentrations prenatally and until weaning, allowing for the evaluation of the role Cu and MT play in developmental neurotoxicity. With the wildtype mice there were 6, 7 and 8 litters for the Cu0, Cu10 and Cu50 conditions and for the MTKO mice there were 7, 8 and 5 litters for the Cu0, Cu10 and Cu50 conditions.

The parental animals were allowed to give birth and after weaning, the offspring were housed with members of the same sex in groups of 2–4 in a Thoren ventilated cage rack in plastic cages with corn cob bedding at 22 ± 2 °C with a 12:12 day:night lighting cycle. All of the mice were provided the same rodent chow and water. Once learning was assessed in the mice and they had reached an age of 120 days, they were euthanized. Prior to testing, four of the knockout mice experienced seizures, after which one mouse died. It is believed that vibrations felt from a moving cart triggered these seizures. Throughout the experiment, animals were handled with care in accordance with an approved animal protocol and institutional and federal animal care guidelines.

### Radial-arm maze

When the offspring had reached 50 days of age, they were tested for the effects of the metallothionein deletion (MTKO) and the various Cu dosage treatments on spatial learning and memory measured by radial-arm maze performance. More specifically, the wild type mouse group exposed to 0 mg/ml of CuSO_4_ *in utero* served as a control representative of normal neurobehavioral development and later learning and memory abilities. The wild type groups administered 5 and 10 mg/ml of CuSO_4_ *in utero* allowed for the observation of Cu’s effects on neurobehavioral development. The MT-1/MT-2- knockout mice that received no prenatal Cu treatment were tested for the effects of metallothionein deletion on radial-arm maze acquisition. Lastly, the MT knockout mice prenatally exposed to 5 or 10 mg/ml of CuSO_4_ were tested in order to demonstrate the combined effects of metallothionein gene deletion and Cu toxicity on the development of neurobehavioral function.

The maze was made from wood and painted black with a center platform 12 cm in diameter. Eight arms (24 × 4 cm) extended from the center and the maze was elevated 25 cm above the floor. The radial-arm maze was located in a room with extra-maze visual cues. Food cups were located at the end of each arm and were baited with a small piece of sweetened cereal (Kellogg’s Froot Loops^©^) was placed in order to serve as bait. Prior to testing on the radial-arm maze, the mice were adapted to handling (two sessions). Afterward, the mice, while restricted to the center of the maze, were exposed to the food reinforcements in order to insure the consumption of the reinforcements (two sessions for all mice except cohort VI which underwent 4 sessions). Thereafter, spatial learning and memory were assessed using the win-shift task in which each arm was baited at the beginning of each session. Each arm entry is rewarded only once since the baits are not replaced. In this way, the animal must remember where it has gone earlier in the session in order to know where to go later. The animals were food restricted once the behavioral testing started.

Prior to testing in the radial-arm maze, each mouse was placed in the center of the maze for 10 s while enclosed in a topless and bottomless opaque cylinder 12 cm in diameter and 10 cm high. The timed session began once the cylinder had been lifted and the mouse was free to explore the maze. When all four paws entered an arm, it was recorded as an arm choice. Each session lasted until the mouse entered all eight arms or when 300 s had elapsed. Choice accuracy was measured as the number of correct arm entries made before an error, or Entries to Repeat. If during a session, a mouse entered only four arms total or less without repeating the session was not included in analysis of choice accuracy due to insufficient information on which to base an accuracy score. Response latency was expressed as the average time in seconds per arm entry, which was calculated by dividing total session length by the total number of arm entries made. If a mouse did not enter one arm maze throughout an entire session, the response latency was recorded as 300 s. 18 sessions were completed for each mouse.

### Neurochemical analysis

Once the mice had been euthanized, their cerebellum, brain stem, midbrain, hippocampus, striatum, anterior cortex, and posterior cortex were surgically isolated. The brain tissue samples were placed in a 0.1 N Perchloric Acid/100 μM EDTA solution of a 10X volume/tissue weight concentration. The tissue and solution combination was homogenized via an ultrasonic tissue homogenizer. In order to remove solid cellular particulate matter, the homogenate underwent column purification. The brain samples were diluted afterward 25X with purified water and norepinephrine, dopamine, serotonin, 3,4-Dihydroxyphenylacetic acid (DOPAC) and 5-Hydroxyindoleacetic acid (5-HIAA) concentrations were determined with High-performance liquid chromatography (HPLC) and calculated per mg of protein.

The HPLC system used consisted of an isocratic pump (model LC1120, GBC Separations) a Rheodyne injector (model 7725i) with a 20-μl PEEK loop, and an INTRO amperometric detector (Antec Leyden). The electrochemical flow cell (model VT 03, Antec Leyden) had a 3 mm glassy carbon working electrode with a 25 μm spacer, and an Ag/AgCl reference electrode. The cell potential was set at 700 mV. The signal was filtered with a low pass in-line noise killer, LINK (Antec Leyden) set at a 14 s peak width and a cut off frequency of 0.086 Hz. The signal is integrated using the EZChrom elite chromatography software (Scientific Software Inc). The injector, flow cell, and analytical column were placed in the Faraday-shielded compartment of the detector where the temperature was maintained at 30 °C.

The stationary phase was a reverse phase column 150 mm × 3.2 mm, with a 3 μm particle size and 120 Å pore size (ESA Scientific). The mobile phase was purchased from ESA Scientific and contained (50 mM H_3_PO_4_, 50 mM citric acid, 100 mg/L 1-octanesulfonic acid (sodium salt), 40 mg/L EDTA, 2 mM KCl and 3 % methanol, corrected to pH 3.0 with NaOH). The mobile phase was continually degassed with a Degasys Populaire on-line degasser (Sanwa Tsusho Co. Ltd.) and delivered at a flow rate of 0.50 ml/min.

### Statistical analysis

Behavioral and neurochemical data were assessed by variance analysis. The between subjects factors were genotype, sex, and Cu treatment for the behavioral as well as the neurochemical analyses. Repeated measures for the behavioral data were session blocks. The dependent measures for the win-shift radial-arm maze task were entries to repeat (the number of correct entries made before the first error) and response latency (average time spent during each arm entry). For neurochemical measurements the neurotransmitter levels per mg of protein were the data analyzed. Neurotransmitter turnover is a function of transmitter level divided by metabolite level. The data were presented as percent of controls for clarity. The raw data for the measures were the basis for statistical analysis. For graphic presentation the data were normalized to percent of control to facilitate comparison of treatment effects in different brain areas and with different neurotransmitters. The threshold for significance was *p* < 0.05. In addition, interactions with *p* < 0.10 were reexamined after separation of the interactive factors (Snedecor & Cochran, 1989). Any significant interactions found were followed up by analyses of the simple main effects of each factor at each of the repeated measures (Keppel, 1973). The threshold for significance for these simple main effects was *p* < 0.05. The 50ug/L Cu exposure in the drinking water caused significant (*p* < 0.001) hypodipsia compared with controls with an average 27.5 % reduction in water consumption. Therefore, the data from this group was removed from the statistical analyses since the effects of this level of high copper could not be differentiated from the effects of hypodipsia. The lower 10ug/L Cu exposure did not cause significant hypodipsia relative to controls.

## Results

### Water consumption and clinical effects

Because the copper was added to the drinking water it was important to measure the consumption to determine the dose administered and whether the copper in the water affected the amount of water consumed. There was a significant main effect of copper treatment on water consumption (*F*(2,62) = 24.45, *p* < 0.005) The Cu0 dams consumed a mean of 3.9 ± 0.2 ml of water per day, the Cu10 dams averaged 3.7 ± 0.2 ml of water per day and the Cu50 dams averaged 2.9 ± 0.2 ml of water per day. Because of the significant (*p* < 0.001) hypodipsia, 26 % decreased from control rates, caused by the Cu50 condition, this treatment group was eliminated from the statistical analyses because direct effects of this concentration of copper on development would be confounded with the effects secondary to hypodipsia of the dams.

### Radial-arm maze learning

**Fig. 1:**
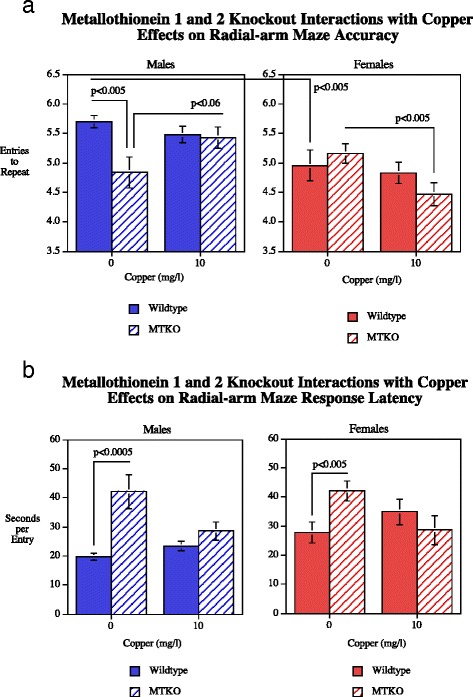
**a**-**b** Radial-arm maze choice accuracy, entries to repeat (mean ± sem). The Genotype x Session Block (*p* < 0.05) prompted simple main effects tests which showed that the MTKO mice had significantly (*p* < 0.0005) worse performance during sessions 1–6. The Genotype x Cu interaction (*p* < 0.08). prompted simple main effects tests which showed that Cu50 significantly (*p* < 0.05) impaired choice accuracy during sessions 13–18 in WT but not MTKO mice. During sessions 1–6 the MTKO mice in the Cu0 and Cu50 treatment groups were significantly worse than the wildtype mice in the same treatment groups. Tobacco extract and nicotine effects on response latencRadial-arm maze response latency Number of rats/condition: Wildtype Cu0 males = 16, Wildtype Cu0 females = 13; Wildtype Cu10 males = 23; Wildtype Cu10 females = 13; MTKO Cu0 males = 11; MTKO Cu0 females = 19; MTKO Cu10 males = 10; MTKO Cu10 females = 15

Radial-arm maze choice accuracy and response latency showed significant effects (Fig. 1a-b). The genotype main effect was significant (*F*(1112) = 4.29, *p* < 0.05) with the MTKO mice performing significantly less accurately than wildtype mice (WT = 5.30 ± 0.09 and MTKO = 4.95 ± 0.11). The effect of sex was significant (*F*(1112) = 15.38, *p* < 0.0005). Males averaged 5.42 ± 0.09 and the MTKO mice averaged 4.95 ± 0.10). The session block main effect was significant (*F*(2224) = 45.58, *p* < 0.0005). The significant Cu x sex interaction (*F*(1112) = 5.18, *p* < 0.025) prompted simple main effects tests of copper in each sex. Females showed a significant choice accuracy impairment caused by Cu10 (*p* < 0.05), whereas no effect was seen in males. The significant genotype x sex x Cu interaction (*F*(1112,) = 7.00, *p* < 0.01) prompted simple main effects tests which showed that only the MTKO females showed a significant effect of Cu0 vs.Cu10 (*p* < 0.005) with copper significantly reducing entries to repeat in the MTKO female mice (4.47 ± 0.19) relative to MTKO female not given supplemental copper (5.16 ± 0.17). There was a significant main effect of the repeated measure of six session block (*F*(2224) = 65.22, *p* < 0.0005) reflecting the improvement in choice accuracy with training, rising from 4.42 ± 0.08 entries to repeat during sessions 1–6 to 5.29 ± 0.09 during trials 7–12 and 5.71 ± 0.11 during sessions 13–18. The significant ((*F*(2224) = 3.50, *p* < 0.01) session block x sex x copper interaction was followed up by tests of the simple main effects of sex and copper treatment at each of block of sessions. During the initial six training sessions there were no significant effects of sex or copper treatment. During the middle six sessions (7–12) there were significant copper effects in both the males (*p* < 0.05) and females (*p* < 0.005) with copper causing higher entries to repeat in males (Cu0 = 5.44 ± 0.19, Cu10 = 5.81 ± 0.16) and lower entries to repeat in females (Cu0 = 5.30 ± 0.16, Cu10 = 4.53 ± 0.16). By the last six-session block (13–16) there were no significant effects of Cu treatment in either males or females.

**Fig. 2:**
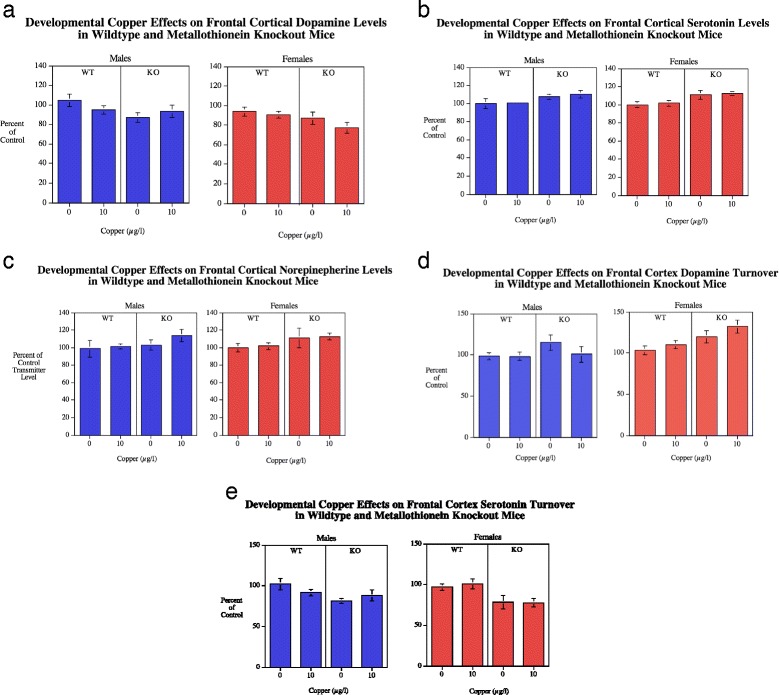
**a**-**c**Frontal cortical DA, 5HT and NE levels (mean ± sem) for each sex, percent of WT Cu0 control mean. There were significant main effect of MTKO increasing frontal cortical DA (*p* < 0.05) 5HT (*p* < 0.005) levels relative to WT mice. With DA levels there was also a significant sex effect with males having higher levels than females (*p* < 0.05). Number of rats/condition: Wildtype Cu0 males = 16, Wildtype Cu0 females = 13; Wildtype Cu10 males = 23; Wildtype Cu10 females = 13; MTKO Cu0 males = 11; MTKO Cu0 females = 19; MTKO Cu10 males = 10; MTKO Cu10 females = 15. **d**-**e** Frontal cortical DA and 5HT turnover (mean ± sem) for each sex, percent of WT Cu0 control mean. MTKO caused a significant (*p* < 0.05) increase in DA turnover relative to WT mice. In contrast the MTKO mice had significantly (*p* < 0.0005) lower 5HT turnover than wildtype controls. Males had significantly (*p* < 0.01) lower DA turnover in the frontal cortex than females

Response latency in the radial-arm maze showed that there were significant main effects of genotype (*F*(1112) = 27.99, *p* < 0.0005) and sex (*F*(1112) = 13.66, *p* < 0.0005). The MTKO mice had significantly longer latencies (41.0 ± 2.3 s/arm entry) than wildtype controls (25.6 ± 1.4 s/arm entry). The sex difference consisted of males (26.7 ± 1.7 s/arm entry) being faster than females (38.6 ± 2.2 s/arm entry). There was no significant main effect of Copper treatment with the latency measure. The interactions of genotype x copper (*F*(1112) = 3.85, *p* = 0.052) and sex x copper (*F*(1112) = 4.31, *p* < 0.05) were followed up by tests of the simple main effects. With regard to copper effects on latency in either of the genotypes, neither the WT nor the KO groups individually showed significant effects of Cu10 on response latency. When broken down by sex there were significant elevations in response latency in both males (*p* < 0.0005) and females (*p* < 0.005) but the addition of copper eliminated these effects (Fig. 2). Session Block (*F*(2224) = 17.91, *p* < 0.0005). There was also a significant (*F*(2224) = 19.32, *p* < 0.0005) interaction of genotype x session block. Tests of the simple main effects of genotype at each session block showed that there was a significantly (*p* < 0.0005) elevated latency in the MTKO group (58.9 ± 6.0) relative to wildtype mice (25.8 ± 1.6) in the first six sessions and during the final six session (wildtype = 25.1 ± 1.6, MTKO = 33.4 ± 1.8, *p* < 0.025), but not in the middle or training. Finally, there was a significant interaction of copper x sex x session block (*F*(2224) = 4.10, *p* < 0.025), but copper treatment was not found to have significant effects on response latency in either males or females in any of the session blocks.

### Neurochemical analyses

#### Frontal cortex

##### Dopamine

With frontal cortical dopamine (DA) concentrations (Fig. 2a) there was a significant (*F*(1113) = 6.01, *p* < 0.05) main effect of genotype with the MTKO mice (276.2 ± 9.6) having significantly lower DA in the frontal cortex than wildtype controls (309.3 ± 8.1). The main effect of sex was also significant (*F*(1113) = 4.03, *p* < 0.05) with males (307.9 ± 9.0) having higher DA levels than females (279.4 ± 8.7). DA turnover in the frontal cortex (Fig. 2d) showed a significant (*F*(1113) = 8.90, *p* < 0.005) main effect of genotype with the MTKO mice (0.169 ± 0.006) having higher DA turnover than wildtype (0.145 ± 0.004). There was also a significant main effect of sex, (*F*(1113) = 7.55, *p* < 0.01) with females (0.168 ± 0.005) having a higher DA turnover rate than males (0.146 ± 0.005). There was an interaction of Cu x sex (*F*(2113) = 3.11, *p* < 0.09), which was followed up by tests of the simple main effects. However the simple main effects of copper in either sex was not significant (Fig. 2b).

##### Serotonin

In the frontal cortex the MTKO mice (139.7 ± 2.4) showed a significant (*F*(1113) = 13.62, *p* < 0.0005) elevation in 5HT levels compared with wildtype controls (126.9 ± 2.1) (Fig. 2b). 5HT turnover in the frontal cortex (Fig. 2e) showed a significant (*F*(1109) = 13.77, *p* < 0.0005) main effect of genotype with a decrease in 5HT turnover in the MTKO mice (1.27 ± 0.05) vs. wildtype mice (1.55 ± 0.04) relative to wildtype controls.

##### Norepinepherine

No significant effect of Cu treatment, genotype or sex was seen with NE in the frontal cortex (Fig. 2c).

#### Posterior cortex

##### Dopamine

**Fig. 3:**
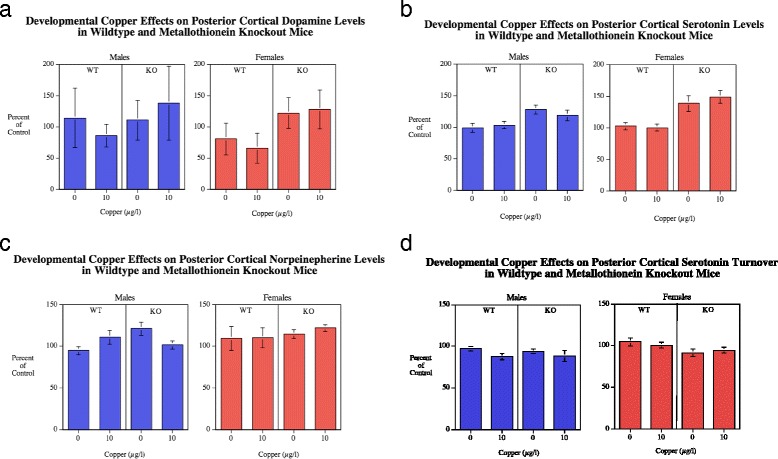
**a**-**c** Posterior cortical DA, 5HT and NE (mean ± sem) for each sex, percent of WT Cu0 control mean. In the posterior cortex there was a significant (*p* < 0.0005) main effect of MTKO increasing 5HT levels relative to WT mice. Number of rats/condition: Wildtype Cu0 males = 16, Wildtype Cu0 females = 13; Wildtype Cu10 males = 23; Wildtype Cu10 females = 13; MTKO Cu0 males = 11; MTKO Cu0 females = 19; MTKO Cu10 males = 10; MTKO Cu10 females = 15. **d** 5HT turnover was significantly (*p* < 0.05) higher in females than males

No significant Cu, genotype and sex main effects on DA levels (Fig. 3a) were seen in the posterior cortex (Fig. 3). DA turnover in the posterior cortex could not be assessed due to problems with analysis of DOPAC for this region.

##### Serotonin

In the posterior cortex the MTKO mice (55.9 ± 2.3) also showed a significant (*F*(1111) = 26.20, *p* < 0.0005) increase in 5HT levels (Fig. 3b) compared with wildtype controls (41.8 ± 1.2) (Fig. 3). There were no other significant main effects or interactions. 5HT turnover in the posterior cortex (Fig. 3d) as in the frontal cortex there was a significant main effect of sex (*F*(1110) = 4.11, *p* < 0.05). The females (0.61 ± 0.01) had significantly greater turnover than males (0.58 ± 0.01).

##### Norepinepherine

In the posterior cortex there were no significant main effect on NE levels (Fig. 3c). There was an interaction of genotype x sex x copper (*F*(2105) = 3.20, *p* < 0.08), which was followed up by tests of the simple main effects of copper in each genotype for each sex. However, none of the simple main effects was significant.

#### Hippocampus

##### Dopamine

**Fig. 4:**
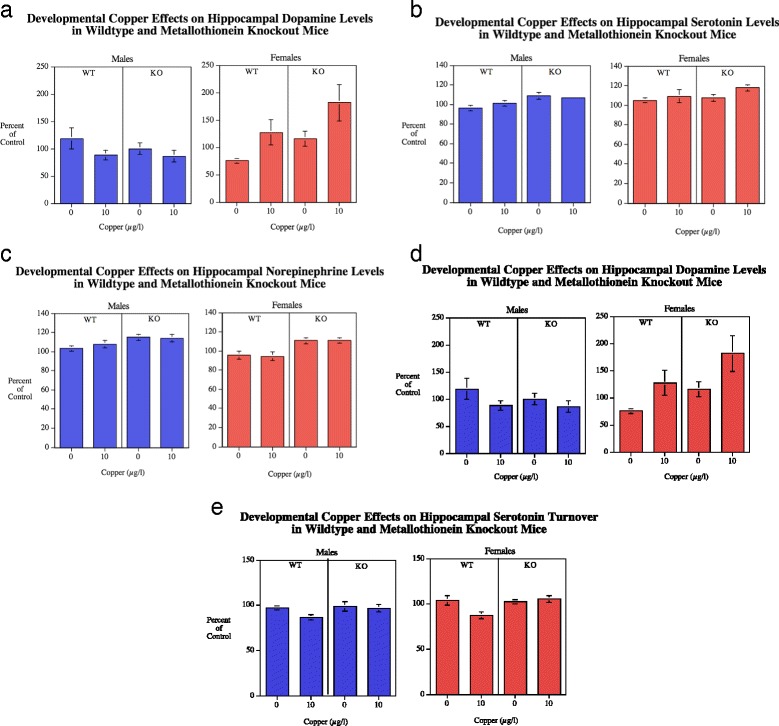
**a**-**c** Hippocampal DA, 5HT and NE (mean ± sem) for each sex, percent of WT Cu0 control mean. There was a significant (*p* < 0.05) main effect of sex on DA levels with females greater than males. With genotype comparisons female MTKO mice had increased DA relative to WT in females. NE (*p* < 0.0001) and 5HT (*p* < 0.005) levels. Number of rats/condition: Wildtype Cu0 males = 16, Wildtype Cu0 females = 13; Wildtype Cu10 males = 23; Wildtype Cu10 females = 13; MTKO Cu0 males = 11; MTKO Cu0 females = 19; MTKO Cu10 males = 10; MTKO Cu10 females = 15. **d**-**e** Hippocampal DA turnover (mean ± sem) for each sex, percent of WT Cu0 control mean. DA turnover was significantly decreased by Cu10 (*p* < 0.025) in females. Hippocampal 5HT turnover was decreased by copper in the WT (*p* < 0.0005) but not MTKO mice. The MTKO mice had significantly (*p* < 0.01) greater 5HT turnover than wildtype

There was no significant main effect of genotype (Fig. 4a). The main effect of sex was significant (*F*(1113) = 4.28, *p* < 0.05) with females (17.7 ± 1.6) having higher hippocampal DA levels than males (13.7 ± 0.9). The genotype x sex (*F*(1113) = 5.10, *p* < 0.05) and sex x Cu (*F*(2113) = 10.06, *p* < 0.005) interactions were followed up by tests of the simple main effects. They showed significant MTKO-induced increase in females (*p* < 0.01) increasing DA levels (wildtype = 14.4 ± 1.9, MTKO = 20.3 ± 2.4), but not in males. There was a significant effect of Cu10 μg/l (21.7 ± 2.8) increasing levels in females relative to female controls (13.8 ± 1.2). No effect was seen in males. With DA turnover (Fig. 4d) the main effect showed that Cu significantly (*F*(2,74) = 5.65, *p* < 0.05) decreased DA turnover with the controls averaging 0.80 ± 0.04 and the Cu10 group 0.66 ± 0.03 (Fig. 4b). Significant interactions were seen with sex x Cu (*F*(1,74) = 9.24, *p* < 0.005) and genotype x sex (*F*(1,74) = 6.25, *p* < 0.05). The simple main effects tests showed that females had a significant (*p* < 0.0005) reduction in DA turnover (0.57 ± 0.03) relative to vehicle control (0.84 ± 0.05, whereas there was no significant effect in males. The tests of simple main effects of genotype for each sex did not detect any significant effects.

##### Serotonin

There was a significant (*F*(1112) = 7.86, *p* < 0.005) main effect of genotype on 5HT levels (Fig. 4b), with MTKO mice (299.4 ± 4.5) having elevated hippocampal 5HT levels compared with wildtype mice (277.3 ± 5.3). The main effect of sex was significant (*F*(1112) = 6.03, *p* < 0.05) with females (297.9 ± 5.8) having higher hippocampal 5HT levels than males (277.3 ± 4.3). With 5HT turnover (Fig. 4e) the main effect of genotype was significant (*F*(1112) = 7.31, *p* < 0.01) with MTKO mice (0.659 ± 0.012) having higher hippocampal 5HT turnover than wildtype (0.602 ± 0.012). The Cu main effect showed a significant decrease (*F*(1112) = 6.13, *p* < 0.05) with the controls averaging 0.653 ± 0.011 and the Cu10 group 0.605 ± 0.013. The significant genotype x Cu interaction (*F*(1112) = 7.19, *p* < 0.01) was followed up by tests of the simple main effects. These showed with the wildtype mice showed a significant (*p* < 0.0005) effect of copper decreasing serotonin turnover (Cu0 = 0.650 ± 0.017, Cu10 = 0.565 ± 0.015) whereas there was no significant effect of copper in the MTKO mice (Fig. 4b).

##### Norepinepherine

There was a significant (*F*(1113) = 20.57, *p* < 0.0005) main effect of genotype (Fig. 4c), with the MTKO mice (191.5 ± 2.8) having higher hippocampal NE levels than the wildtype mice (173.2 ± 3.5). There was a significant main effect of sex (*F*(1113) = 6.87, *p* < 0.05) with males (186.1 ± 3.2) having higher hippocampal NE levels than females (177.0 ± 3.6).

#### Striatum

##### Dopamine

**Fig. 5:**
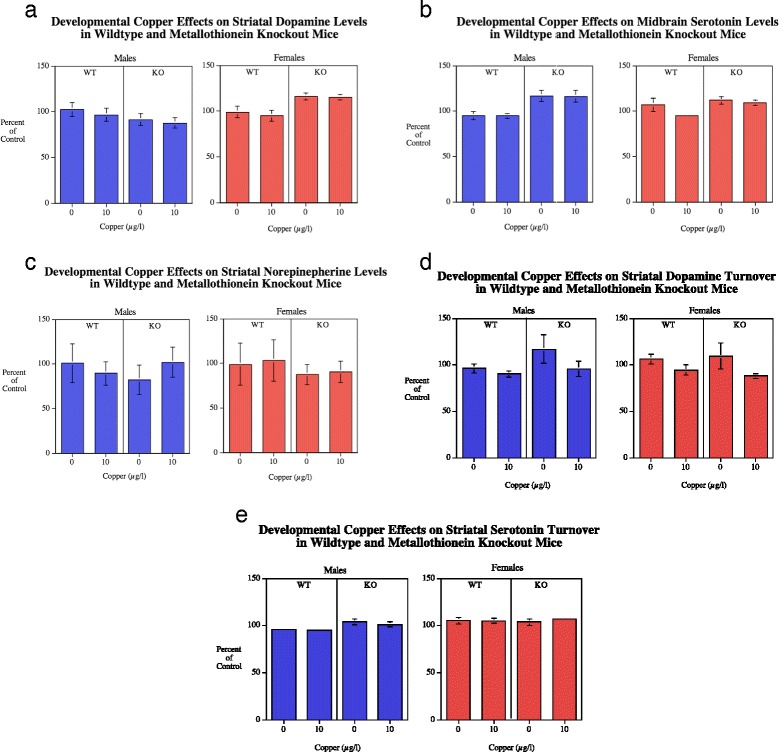
**a**-**c** Striatal NE, DA and 5HT (mean ± sem) for each sex, percent of WT Cu0 control mean.. The MTKO males had significantly(*p* < 0.005) higher 5HT level than WT males. Number of rats/condition: Wildtype Cu0 males = 16, Wildtype Cu0 females = 13; Wildtype Cu10 males = 23; Wildtype Cu10 females = 13; MTKO Cu0 males = 11; MTKO Cu0 females = 19; MTKO Cu10 males = 10; MTKO Cu10 females = 15. **d**-**e** Striatal DA and 5HT turnover (mean ± sem) for each sex, percent of WT Cu0 control mean. There was a significant (*p* < 0.05) decrease in DA turnover caused by Cu10

There was not a significant (*F*(1112) = 10.69, *p* < 0.005) main effect of genotype (Fig. 5a). With DA turnover there was a significant main effect of Cu treatment (*F*(1112) = 6.19, *p* < 0.05) with lowering of striatal DA turnover (Fig. 5d) by Cu10 (0.102 ± 0.003) relative to untreated controls (0.118 ± 0.006).

##### Serotonin

There was a very significant (*F*(1112) = 10.69, *p* < 0.005) elevation of striatal serotonin (Fig. 5b) in the MTKO mice (79.83 ± 1.15) relative to wildtype controls (70.44 ± 2.35) (Fig. 5a). There were no other significant main effects or interactions. With 5HT turnover there was a significant main effect of sex (*F*(1112) = 10.02, *p* < 0.005) with females (0.434 ± 0.006) having higher turnover rates than males (0.410 ± 0.005) (Fig. 5e). There was an interaction of genotype x sex (*F*(2112) = 3.20, *p* < 0.08) that prompted analyses of the simple main effects. MTKO males (0.424 ± 0.008) showed a significantly (*p* < 0.05) increased striatal serotonin turnover relative to wildtype males (0.394 ± 0.006). In contrast, no effects were seen in females.

##### Norepinepherine

There were no significant effects detected concerning NE levels in the striatum (Fig. 5c).

#### Midbrain

##### Dopamine

**Fig. 6:**
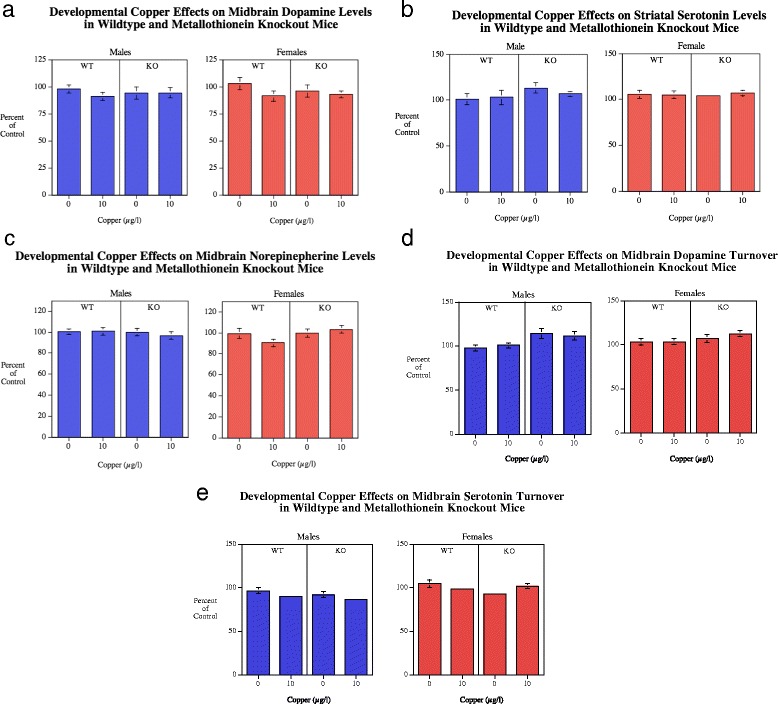
**a**-**c** Midbrain DA (mean ± sem) for each sex, percent of WT Cu0 control mean. With 5HT there was a significant (*p* < 0.0005) main effect of genotype with higher levels in the MTKO vs. WT mice. Number of rats/condition: Wildtype Cu0 males = 16, Wildtype Cu0 females = 13; Wildtype Cu10 males = 23; Wildtype Cu10 females = 13; MTKO Cu0 males = 11; MTKO Cu0 females = 19; MTKO Cu10 males = 10; MTKO Cu10 females = 15. **d**-**e** Midbrain DA and 5HT turnover (mean ± sem) for each sex, percent of WT Cu0 control mean. DA turnover was significantly (*p* < 0.001) increased in MTKO vs. WT mice. With 5HT turnover, there was a significant (*p* < 0.05) decrease with copper treatment in WT males, but a significant(*p* < 0.05) increase in MTKO females

There were no significant effects on DA in the midbrain (Fig. 6a). With DA turnover there was a significant genotype main effect (*F*(1108) = 12.67, *p* < 0.001). The MTKO mice had higher DA turnover in the midbrain (0.48 ± 0.01) than the wildtype controls (0.44 ± 0.01) (Fig. 6d).

##### Serotonin

There was a highly significant (*F*(1109) = 23.15, *p* < 0.0005) main effect of genotype on midbrain serotonin levels with wildtype controls averaging 408.3 ± 9.0 and the MTKO mice averaging 476.1 ± 10.0 (Fig. 6b). The genotype x sex interaction (*F*(1109) = 3.57, *p* < 0.07) prompted tests of the simple main effects of genotype in each sex. Both male (*p* < 0.0005) and female (*p* < 0.05) MTKO mice showed significant elevations is serotonin relative to wildtype controls. With 5HT turnover (Fig. 6e) there were significant main effects of genotype (*F*(1109) = 4.52, *p* < 0.05) with the MTKO mice (0.536 ± 0.008) having lower turnover than the wildtype mice (0.552 ± 0.009) (Fig. 6e). There was a significant main effect of sex (*F*(1109) = 17.26, *p* < 0.0005) with males having lower turnover (0.525 ± 0.008) than females (0.566 ± 0.009). There were significant two-way interactions of genotype x Cu (*F*(2109) = 4.46, *p* < 0.05) and sex x copper (F1109) = 3.98, *p* < 0.05). There was also a three-way interaction of genotype x sex x copper (*F*(1,109) = 3.29, *p* < 0.08) prompting simple main effects. So the simple main effects tests were made at this level. The wildtype males showed a significant decrease (*p* < 0.05) with copper treatment and MTKO females showed a significant (*p* < 0.05) increase with copper treatment.

##### Norepinepherine

There were no significant effects on NE levels in the midbrain (Fig. 6c).

#### Brainstem

##### Dopamine

**Fig. 7:**
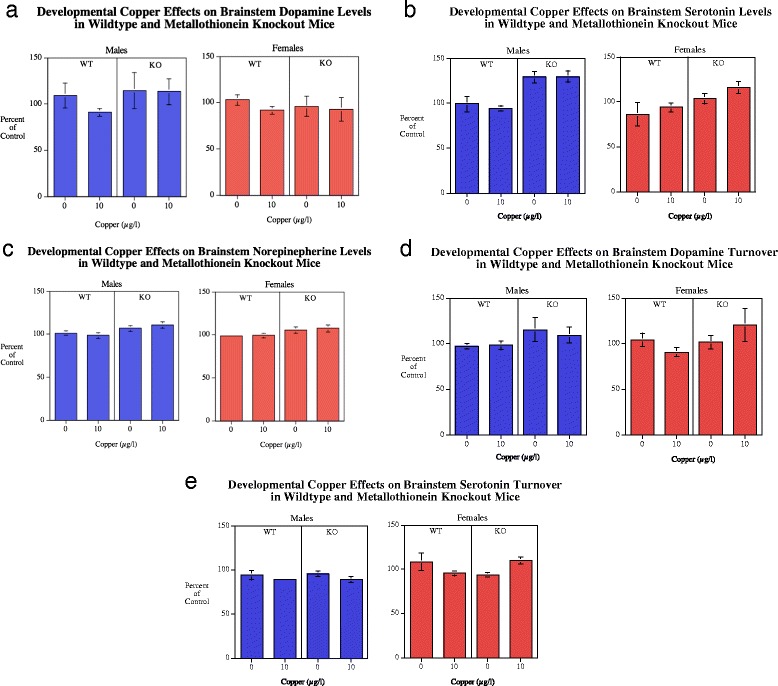
**a**-**c** Brainstem DA, 5HT and NE (mean ± sem) for each sex, percent of WT Cu0 control mean. There was a significant main effect of genotype with DA levels (*p* < 0.05) with MTKO causing an overall increase. A robust effect was seen with significantly (*p* < 0.0005) higher 5HT levels in male MTKO mice relative to male WT. NE levels in MTKO mice were significantly (*p* < 0.005) higher than WT regardless of sex. Number of rats/condition: Wildtype Cu0 males = 16, Wildtype Cu0 females = 13; Wildtype Cu10 males = 23; Wildtype Cu10 females = 13; MTKO Cu0 males = 11; MTKO Cu0 females = 19; MTKO Cu10 males = 10; MTKO Cu10 females = 15. **d**-**e** Brainstem DA and 5HT turnover (mean ± sem) for each sex, percent of WT Cu0 control mean. With DA turnover there was a significant (*p* < 0.05) main effect of genotype with MTKO mice having higher turnover than WT mice. With 5HT turnover there was a significant (*p* < 0.05) decrease caused by Cu10 in female WT mice, but a significant (*p* < 0.005) increase in female MTKO mice. There was a significant (*p* < 0.001) sex effect with males having lower turnover than females

There was a significant main effect of genotype (*F*(1110) = 4.45, *p* < 0.05) with the MTKO mice (31.2 ± 1.9) having higher dopamine levels in the brainstem than wildtype controls (26.7 ± 1.1) (Fig. 7a). With DA turnover there was a significant (*F*(1,90) = 5.65, *p* < 0.05) effect of genotype with regard to dopamine turnover in the brainstem (Fig. 7d). The MTKO mice averaged 0.72 ± 0.04 while the wildtype controls averaged 0.64 ± 0.02. There was also an interaction of genotype x sex x copper (*F*(1,90) = 2.81, *p* = 0.097), however none of the simple main effects of copper treatment in either genotype of either sex were significant.

##### Serotonin

As in the other brain regions there was a very significant (*F*(1112) = 22.94, *p* < 0.0005) effect of genotype on serotonin levels in the brainstem with MTKO mice (386.2 ± 10.3) having higher levels than wildtype mice (313.4 ± 10.6) (Fig. 7b). There was a genotype x sex interaction (*F*(1112) = 4.32, *p* < 0.05) that prompted tests of the simple main effects. Males (*p* < 0.0005) but not females (*p* = 0.059) showed significantly elevated brainstem serotonin levels. With 5HT turnover (Fig. 7e) there was a significant (F1112) = 11.66, *p* < 0.001) main effect of sex with the males (0.64 ± 0.01) showing a lower turnover than females (0.70 ± 0.02). There were also significant interactions of genotype x Cu (*F*(1,112) = 5.63, *p* < 0.05) and genotype x Cu x sex (*F*(2112) = 7.28, *p* < 0.01). With the wildtype mice the Cu10 dose caused a significant (*p* < 0.05) decrease in serotonin turnover, but this was not seen in the MTKO mice (Fig. 7e). The simple main effects within each sex showed no significant effect with males. However with females the wildtype mice showed a significant decrease in serotonin turnover with the Cu10 (*p* < 0.05), while the MTKO female mice showed that Cu10 dose caused a significant (*p* < 0.005) increase in serotonin turnover relative to MTKO female mice not given supplemental Cu. No significant effects were seen in males.

##### Norepinepherine

There was a significant (*F*(1112) = 10.95, *p* < 0.005) genotype effect with the MTKO (409.4 ± 7.2) mice having higher brainstem NE levels than wildtype mice (378.6 ± 5.8) (Fig. 7c).

##### Correlation of memory and neurochemical measures

The relationship between choice accuracy on the radial-arm maze (mean entries to repeat sessions 1–18) and serotonin, dopamine and norepinepherine measures were determined. Serotonin measurements in several brain regions showed significant correlations with memory performance. There was a significant negative correlation of hippocampal (*p* < 0.001, *r* ^2^ = 0.099) serotonin levels with radial-arm maze choice accuracy, with lower concentrations associated with better choice accuracy. In the striatum, serotonin turnover had a significant (*p* < 0.0005, *r* ^2^ = 0.112) negative correlation with radial-arm maze choice accuracy with lower turnover rates associated with better choice accuracy. Similar significant correlations were seen with serotonin turnover in the midbrain (*p* < 0.05, *r* ^2^ = 0.039) and the brainstem (*p* < 0.05, r2 = 0.043). Dopamine levels in the frontal cortex had a positive correlation with better choice accuracy (*p* < 0.05, *r* ^2^ = 0.041). Dopamine turnover in the frontal cortex had a negative correlation with radial-arm maze choice accuracy (*p* < 0.01, *r* ^2^ = 0.064).

## Discussion

Copper added to the drinking water produced significant neurochemical effects in the offspring in all subcortical areas (Table 1). Effects were seen with DA and 5HT but not NE. Additional copper increased DA turnover in the posterior cortex in females. In contrast, additional copper decreased DA turnover and increased DA levels in the hippocampus in females. In the striatum additional copper decreased DA turnover regardless of sex. 5HT turnover was significantly decreased by copper in the hippocampus and brainstem of WT but not MTKO mice. In the midbrain, copper had opposite effects on 5HT turnover in males (decrease) and females (increase). In the brainstem copper treatment had opposite effects in female WT (decreased) and female MTKO (increased) mice.

**Table 1:** Summary of copper, genotype and sex effects on dopamine, serotonin and norepinephrine

	Copper	MTKO	Sex
Frontal Cortex			
DA Level		−11 % MTKO < WT	+10 % Males > Females
DA Turnover		+17 % MTKO > WT	−13 % Males < Females
5HT Level		+10 % MTKO > WT	
5HT Turnover		−17 % MTKO < WT	
NE Level			
Posterior Cortex			
DA Level			
5HT Level		+34 % MTKO > WT	
5HT Turnover			−6 % Males < Females
NE Level			
Hippocampus			
DA Level	+56 % Cu > Con Females	+41 % MTKO > WT Females	
DA Turnover	−31 % Cu < Con Females		
5HT Level		+8 % MTKO > WT	−7 % Males < Females
5HT Turnover	−13 % Cu < Con in WT		
NE Level		+11 % MTKO > WT	+5 % Males > Females
Striatum			
DA Level			
DA Turnover	−14 % Cu < Con		
5HT Level		+13 % MTKO > WT	
5HT Turnover			
NE Level			
Midbrain			
DA Level			
DA Turnover		+10%MTKO > WT	
5HT Level		+17 % MTKO > WT	
5HT Turnover	−7 % Cu < Con WT Males		
	+10 % Cu > Con MTKO Females		
NE Level			
Brainstem			
DA Level		+17 % MTKO > WT	
DA Turnover		+13 % MTKO > WT	
5HT Level		+35 % MTKO > WT Males	
5HT Turnover	−12 % Cu < Con female WT		
	+17 % Cu > Con Female MTKO		
NE Level		+8 % MTKO > WT	

The more prominent neurochemical effects of additional copper in females may be related to the selective effects of additional copper impairing radial-arm maze accuracy in females. In particular the sex-selective effects of additional copper on DA in the hippocampus of female rats may be relevant to the impairment since hippocampal DA innervation has been shown to be important for spatial memory function [26]. Also relevant to the sex-selective copper induced radial-arm maze impacts may be copper effects on serotonin in more caudal parts of the brain. In the midbrain there were opposite effects of copper addition on 5HT turnover with increased turnover in males and decreased turnover in females which matches the improved radial-arm maze accuracy in males and impaired performance in females given added copper. Even more to the point was the finding of opposite significant effects of added copper decreasing 5HT turnover in female WT mice and significantly increased 5HT turnover in female MTKO mice. This matched the selective radial-arm maze accuracy impairment of MTKO female mice by supplemental copper.

Knockouts of genes for metallothionein 1 and 2 (MTKO) produced significant impairments of radial-arm maze choice accuracy [2, 3]. The effect was seen principally in males. In rodents as well as other mammals, males are often seen to perform more accurately than females on spatial tasks like the radial-arm maze [27, 28]. The normal male-female difference with males having higher accuracy than females in the spatial radial-arm maze was also seen in wildtype mice in the current study. Male MTKO mice had a choice accuracy impairment, which brought them down to WT female levels. Moderate copper supplementation during development caused significant effects on radial-arm maze choice accuracy. Cu10 reversed the memory impairment caused by MTKO. In contrast, this same level of copper supplementation caused impaired memory performance in MTKO females. The net effect of the moderate Cu supplementation was to re-establish the sex difference in spatial memory absent in MTKO mice without Cu supplementation.

There were also a variety of persistent neurochemical effects in monoaminergic neurotransmitter systems. Previously, we found that serotonin levels were substantially higher in the frontal cortex of MTKO mice than wildtype (WT) controls [2]. The current study replicated this finding of increased serotonin levels in the in the frontal cortex of MTKO mice. It also considerably extended the finding to demonstrate in a novel finding significantly higher serotonin levels of the posterior cortex, hippocampus, striatum, midbrain and brainstem of MTKO mice. In addition, serotonin turnover was significantly lowered by MTKO in the frontal cortex. Dopamine and norepinepherine were altered by MTKO as well, but in much more modest ways. Dopamine turnover was increased by MTKO relative to WT in the frontal cortex, midbrain and brainstem. Norepinepherine was increased by MTKO relative to WT just in the hippocampus and brainstem.

Cu supplementation had effects on behavioral function and neurochemical indices. Several effects were seen, regardless of genotype of the mice. Cu10 caused a decrease in hippocampal and striatal dopamine turnover. In the hippocampus this was restricted to females, as was the substantial copper induced increase in dopamine levels. MTKO modified reaction to Cu supplementation in many of the neurochemical indices. In WT mice Cu10 caused a significant decrease in hippocampal serotonin turnover. In the brainstem the Cu10 supplementation significantly decreased serotonin turnover in wildtype, but not MTKO mice. In wildtype mice, Cu supplementation during development caused a significant decline in dopamine and norepinepherine in the midbrain and dopamine in the frontal cortex. These effects were blocked by MTKO.

There were sex effects on DA markers and differential effects of Cu on DA in males and females. Overall, males had higher DA concentrations in the frontal cortex, whereas females had higher DA concentrations in the hippocampus. In the hippocampus, the higher DA levels in females were driven by the MTKO females, which were higher than wildtype females, which did not differ from males or either genotype. Also in the hippocampus, Cu10 increased DA levels in females, but not males. In the striatum, MTKO females but not males showed higher DA levels. In the posterior cortex, there was a sex interaction with genotype in which in which female MTKO mice had higher DA turnover than wildtype females whereas no effect was seen in males. In addition, there was an interaction of sex with Cu in which females showed an increase in DA turnover in the posterior cortex after Cu10 treatment, but no effects were seen with the higher dose or with either dose in males. The lower dose of Cu (Cu10) but not the higher dose also decreased hippocampal DA turnover.

Decreased serotonin levels in all regions in MTKO mice were the most pervasive effect of the entire study. As seen previously [2]. In the striatum this elevation in serotonin in MTKO mice was limited to females, but in the other areas it was seen in both sexes. Serotonin turnover was decreased in the frontal and posterior cortices and midbrain of MTKO mice compared with wildtype mice.

Females had higher serotonin concentrations than males in the posterior cortex . They had higher serotonin turnover than males in the hippocampus. Females had larger effects of MTKO than males with regard to serotonergic systems. The MTKO increased serotonin levels in the all areas tested, particularly in the posterior cortex and brainstem. Cu treatment also affected serotonin systems but in a complex fashion. Cu significantly decreased hippocampal serotonin turnover but MTKO mice were not significantly affected. In the midbrain wildtype males showed a significant decrease in serotonin turnover caused by Cu. In contrast, MTKO females showed increased serotonin turnover with Cu10 treatment. In the brainstem the wildtype mice the Cu10 caused a decrease in serotonin turnover. The simple main effects within each sex showed no significant effect with males. However with females the wildtype mice showed a decrease in serotonin turnover with the Cu10 while the MTKO female mice showed that Cu10 caused an increase in serotonin turnover relative to MTKO female mice not given supplemental Cu.

Norepinepherine was elevated in the hippocampus and brainstem of MTKO mice, though quite modestly. Males in general had higher hippocampal norepinepherine than females. Though this was also a fairly modest effect.

There were significant relationships between regional transmitter levels and memory performance though these were modest. There were inverse correlations between average choice accuracy during radial-arm maze and serotonin systems in several brain areas. The hippocampal and brainstem levels of serotonin showed this inverse correlation with radial-arm maze choice accuracy. Similar inverse correlations were seen with striatal serotonin turnover rates and radial-arm maze choice accuracy. Other studies have found that serotonergic systems in the hippocampus are important for memory. With dopamine systems, only one region showed a significant correlation with radial-arm maze choice accuracy. Dopamine turnover in the frontal cortex had a negative correlation with choice accuracy. Frontal cortical dopamine has been found to be important for memory function [29].

## Conclusions

This study showed that MTKO mice were more susceptible to the neurobehavioral effects of developmental Cu exposure. This effect may be a result of greater retention of Cu and the lack of metallothioneins 1 and 2 to shepherd Cu to organs, including the brain. Future studies should look at the role other steps in Cu metabolism, such as ceruloplasmin have in neurocognitive development [10].

## Abbreviations

5HT: Serotonin
Cu: Copper
DA: Dopamine
MT: Metallothionein
MTKO: Methallothionein Knockout
NE: Norepinepherine
WT: Wildtype
